# Enhanced anti-tumor therapeutic efficacy of DNA vaccine by fusing the E7 gene to BAFF in treating human papillomavirus-associated cancer

**DOI:** 10.18632/oncotarget.16032

**Published:** 2017-03-09

**Authors:** Chao-Chih Wu, Fang-Cih Wu, Yun-Tin Hsu, Yu-Chia Hsiao, Yuh-Cheng Yang, C. Allen Chang, Chih-Long Chang

**Affiliations:** ^1^ Department of Obstetrics and Gynecology, Mackay Memorial Hospital, Taipei City, Taiwan; ^2^ Departmental of Medical Research, Mackay Memorial Hospital, New Taipei City, Taiwan; ^3^ Department of Medicine, Mackay Medical College, Sanchi, New Taipei City, Taiwan; ^4^ Department of Biotechnology and Laboratory Science in Medicine, School of Biomedical Science and Engineering, National Yang-Ming University, Taipei City, Taiwan; ^5^ Department of Biomedical Imaging and Radiological Sciences, School of Biomedical Science and Engineering, National Yang-Ming University, Taipei City, Taiwan

**Keywords:** DNA vaccine, BAFF

## Abstract

B-cell-activating factor (BAFF) belongs to the tumor necrosis factor family that not only stimulates B and T cells but also counteracts immune tolerance. BAFF is also a type II membrane protein, which is secreted through the endoplasmic reticulum (ER)–Golgi apparatus pathway. Fusing an antigen to BAFF might enhance the presentation of major histocompatibility complex class I molecules. These characteristics represent an opportunity to enhance the antitumor effects of DNA vaccines. Therefore, we fused BAFF to human papillomavirus type 16 E7 as a DNA vaccine and evaluated its antitumor effects. We found that this vaccine increased E7-specific CD8^+^ T-cell immune responses, engendered major antitumor effects against E7-expressing tumors, and prolonged the survival of the immunized mice. Interestingly, vaccinating B-cell-deficient mice with BAFF–E7 revealed considerable E7-specific CD8^+^ T-cell immune responses, suggesting that B cells do not contribute to this immune response. Image analysis through confocal fluorescence microscopy revealed that fusing BAFF to E7 targeted the protein to the ER, but not BAFF lacking 128 N-terminal residues that generated a lower number of E7-specific CD8^+^ T cells in the vaccinated mice. Our data indicated that the ER-targeting characteristic of BAFF is the main factor improving the potency of DNA vaccines.

## INTRODUCTION

Developing an effective cancer therapy is a major concern in contemporary medicine. Solid tumors are mainly treated through surgical debulking of tumor masses followed by adjuvant chemotherapy or radiotherapy. Although growing clinical evidences in addition to technical improvements have improved the therapeutic effects of solid cancer treatment, the relapses of these malignant diseases remain a serious problem.

Immunotherapy is a cancer treatment modality that stimulates the body's immune system against tumors. Immunotherapy can specifically destroy tumor cells without harming normal cells by training the self-immune system to recognize cancer antigens. DNA vaccines are a type of cancer vaccine administered by delivering tumor-associated antigen-expressing vectors into antigen-presenting cells [1]. The engineered tumor-associated antigens expressed in these cells are further processed and presented to T cells by major histocompatibility complex (MHC) molecules. In contrast to other vaccine types, DNA vaccines can be efficiently manipulated through molecular cloning techniques and are easy to be stored and delivered [2]. Furthermore, in contrast to conventional protein-based vaccines that can induce only humoral immune responses, DNA vaccines can induce not only CD4^+^ T-cell responses but also CD8^+^ cytotoxic T-cell responses that effectively target tumor-associated antigens expressed inside the cells and presented by MHC class I molecules [3]. Therefore, stimulating immune responses by using tumor antigen-expressed DNA vaccines is a potential cancer treatment modality. Except for tumor antigen-coding vaccines, cytokine-expressing vectors, such as granulocyte-macrophage colony-stimulating factor and interleukin (IL)-2, have been coadministered to enhance the immune stimulation effects of DNA vaccines [4, 5]. Moreover, recombinant DNA vaccines that involve combining tumor antigens with genes that enhance antigen processing or dendritic cell (DC) responses have been developed [6–8]. For example, a DNA vaccine encoding calreticulin fused to human papillomavirus (HPV) E7 genes exhibited enhanced E7-specific CD8^+^ T-cell responses through an enhanced MHC I presentation because of the chaperone effect of calreticulin [9]. Additional DNA vaccines that activate immunity against poor-immune-response-inducing tumor antigens by fusing them to immunogenic microbial proteins were shown to suppress tumor growth [10].

The tumor necrosis factor (TNF) superfamily is a critical mediator of multiple physiological processes [11–13]. Many members of this superfamily are also involved in the regulation of immune systems [14]. Through the binding of ligands to the corresponding receptors, the members of the TNF superfamily mediate either programmed cell death (Fas, TNF, TRAIL, VEGI, and LIGHT) or the proliferation (CD27L, CD49L, OX40L, and 4-1BB), survival (B-cell-activating factor [15] and receptor activator of nuclear factor-kappa B ligand [13]), and differentiation (TNF, RANKL, and death receptor 6) of cells by activating caspase-3, p42/44 mitogen-activated protein (MAP) kinase, c-Jun N-terminal kinase, p38 MAP kinase, and nuclear factor-kappa B. Numerous immune-associated cells express the ligands and receptors of the TNF superfamily [16]. Furthermore, 4-1BBL, OX40L CD27, CD30L, and CD40L are essential for T-cell activation. Other factors such as CD40 and RANKL are involved in the activation of DCs. On the basis of these effects, certain members of the TNF superfamily were used as adjuvants or directly fused to DNA vaccines to enhance various antigen-specific immune responses. A previous study reported that mice coimmunized with CD40 expression vectors and a H5N1 DNA vaccine showed an enhanced production of serum anti-HA antibody and expression level of T-helper 2 cytokines, compared with those immunized with the H5N1 DNA vaccine alone [17]. In another study, 4-1BB, OX40L, and CD70 were fused to target genes of a DNA vaccine, and they were demonstrated to induce strong T-cell activities against antigens [18]. A long-term memory of T cells was elicited by DNA vaccine immunization [19]. All of these studies have indicated that the members of the TNF superfamily are potential candidates as adjuvants for DNA vaccines.

BAFF is mostly expressed by T cells, DCs, monocytes, and macrophages. It plays a crucial role in B-cell survival and maturation [15, 20–22]. It has been also reported to stimulate T cells by costimulating the T-cell receptor-dependent pathway [23]. Studies have shown that coimmunizing multi-trimeric BAFF, and 4-1BB or OX40L, with a HIV-1 Gag DNA vaccine can considerably increase antigen-specific CD4^+^ and CD8^+^ T-cell proliferation [24, 25]. In addition, DNA vaccines constructed by fusing antigens directly to BAFF increased the level of serum antigen-specific antibodies [26]. BAFF, similar to numerous members of the TNF superfamily, is a type II transmembrane protein. BAFF proteins are delivered to the cell membrane through the endoplasmic reticulum (ER)–Golgi pathway. Therefore, antigens fused to BAFF might be forced to get into the ER, increasing the possibility of them being processed and presented by MHC class I molecules to enhance immunogenicity.

DNA vaccines designed by fusing BAFF with antigens can enhance target-specific immune responses. Evaluation of such vaccine construct in enhancing the antitumor effect of tumor-specific DNA vaccines is warranted. Therefore, we conducted an investigation by using a HPV type 16 E7-expressing tumor cell, TC-1, as a tumor model. A BAFF–E7-fused gene was constructed and administered through a gene gun system to evaluate its preventive and therapeutic activities against TC-1 tumor growth. Tumor-specific cytotoxic T cells play a key role against solid tumor growth in cancer immunotherapy. The generation of E7-specific CD8^+^ T cells was detected in mice treated with different vaccine constructs. Moreover, the interaction of DCs, T cells, and B cells was studied by coculturing BAFF–E7-expressed DCs with E7-specific CD8^+^ T cells and normal B cells. The involvement of B cells in generating the E7-specific CD8^+^ T-cell was also examined in B-cell-deficient μMT mice that showed a targeted disruption of the immunoglobulin mu chain gene [27]. In addition, the transmembrane domain of BAFF was truncated and administered to TC-1-bearing mice to investigate the relationship between the cell localization of the BAFF–E7 fusion protein and the immunogenicity of this DNA vaccine. In summary, this study demonstrated the antitumor effect of the BAFF–E7 fusion DNA vaccine, and explored the possible mechanisms of this vaccine. Our results suggest the benefit and potential of this DNA vaccine design in cancer treatment.

## RESULTS

### Significant enhancement of systemic and tumor-infiltrated E7-specific CD8^+^ T-cell immune response was induced by chimeric BAFF–E7 vaccine

**Figure 1:**
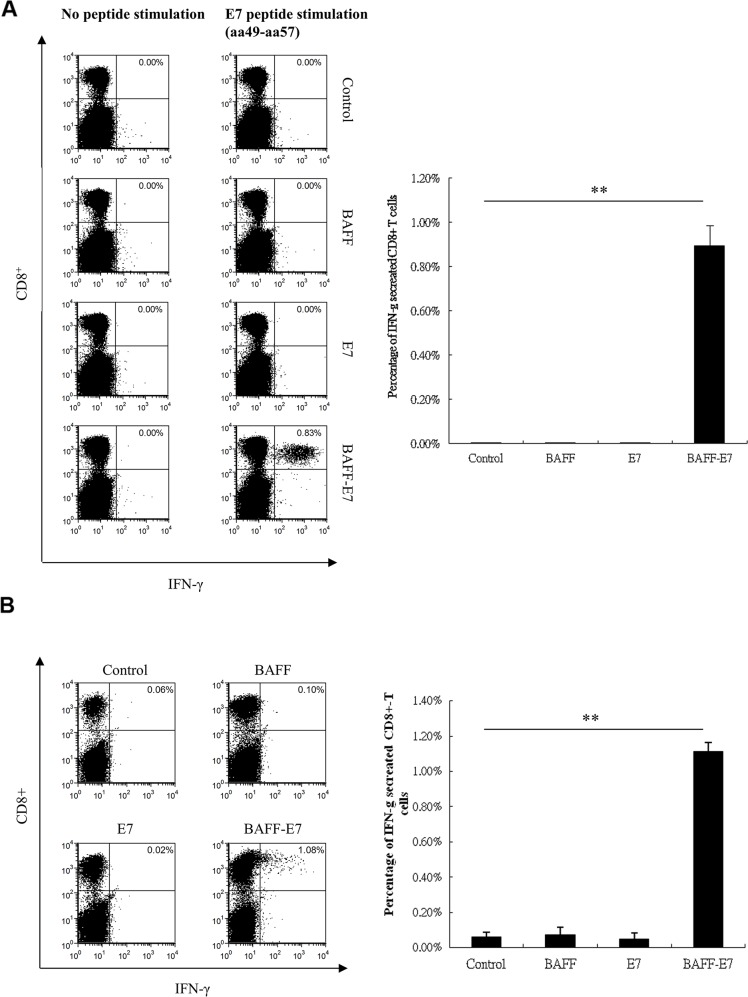
Characterization of the HPV16 E7-specific CD8+ T-cell immune responses in TC-1 tumor-bearing mice vaccinated with the BAFF, E7, or BAFF-E7 DNA vaccine C57BL/6 mice were subcutaneously inoculated with 1 × 10^5^ TC-1 cells. Five days later, tumor-bearing mice were immunized with 2 μg of BAFF, E7, or BAFF-E7 DNA vaccine using a gene gun for total three times at 5-day intervals. Untreated mice served as the control. **(A)** Splenocytes from the different groups of mice were restimulated ex vivo with HPV16 E7 peptide and then were characterized for E7-specific CD8^+^ T cells through flow analysis of intracellular IFN-γ staining cells. Splenocytes without peptide stimulation represented the background control. **(B)** E7-specific CD8^+^ tumor-infiltrated lymphocytes were determined by dissecting and dissociating tumor tissues from each group, followed by intracellular IFN-γ staining and flow analysis. Representative flow data and statistical graphs demonstrated that chimeric BAFF-E7 DNA vaccine increase the percentage of both systemic and local E7-specific CD8^+^ T cell population, but mice vaccinated with pcDNA3.1, BAFF, or E7 did not exceed background levels (p<0.005, BAFF-E7 versus other groups). Error bar of each chart represents the standard error. **p< 0.005.

CD8^+^ T lymphocytes are one of the most crucial effectors in inducing antitumor immunity. We first investigated whether co-administered BAFF and E7 DNA vaccine can induce the generation of E7-specific CD8^+^ T cells. By stimulating splenocytes form vaccinated mice with E7 peptide and analyzing the proportion of IFN-γproducing CD8^+^ T cells, we found that all BAFF, E7 and BAFF mixed E7 vaccine cannot induced E7-specific CD8^+^ T cell generation (Supplementary Figure 1). We further investigated whether the chimeric BAFF–E7 DNA vaccine can induce a specific CD8^+^ T-cell immune response *in vivo*. Splenocytes were harvested from the spleens of the vaccinated mice, and were used to assess the quantity of E7-specific CD8^+^ T cells produced by pcDNA3.1, BAFF, E7, and the chimeric BAFF–E7 DNA vaccine by conducting intracellular cytokine staining, a sensitive functional assay for measuring IFN-γ production at the single-cell level. The representative experiments are shown in Figure 1A. Vaccination with the chimeric BAFF–E7 DNA vaccine exhibited the highest frequency of E7-specific CD8^+^/IFN-γ^+^ double-positive T cells, whereas the proportion of E7-specific CD8^+^/IFN-γ^+^ double-positive T cells in the mice vaccinated with pcDNA3.1, BAFF, or E7 did not exceed background levels (P < 0.005). These findings indicate that vaccination with the chimeric BAFF–E7 DNA vaccine increased the percentage of systemic E7-specific CD8^+^ T lymphocytes.

It has been documented that increasing number of tumor-infiltrated CD8^+^ T cells positively correlated with antitumor effects in solid tumor. We performed intracellular cytokine staining analyzed by flow cytometry to quantify the number of infiltrating CD8^+^ T cells that produced IFN-γ within the tumors from the vaccinated mice. As shown in Figure 1B, an increased percentage of tumor-infiltrated CD8^+^ T lymphocytes was detected in the mice vaccinated with chimeric BAFF–E7 (1.08%) compared with those observed in mice vaccinated with pcDNA3.1, BAFF, or E7 DNA (P < 0.005).

### Chimeric BAFF–E7 DNA vaccine exhibited both preventive and therapeutic effects against the growth of TC-1 tumors

**Figure 2:**
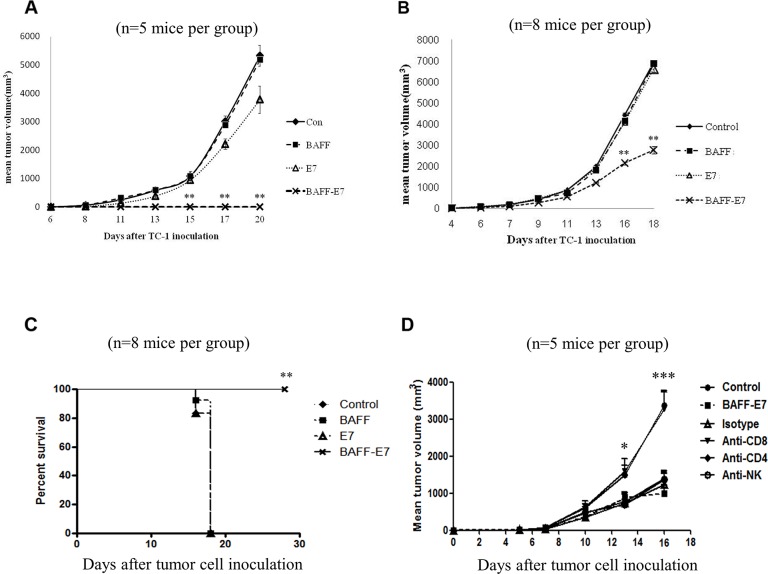
Protective and therapeutic effects of the BAFF–E7 DNA vaccine **(A)** C57BL/6 mice (five per group) were immunized with 2 μg of different DNA constructs three times at 5-day intervals. Five days after the final vaccination, mice were subcutaneously injected with TC-1 tumor cells (10^5^/mouse). Protective effects of the DNA vaccines were shown by the tumor rejection. Tumor volume of mice treated by BAFF-E7 DNA vaccine was significantly smaller than that of other groups (p<0.005, BAFF-E7 versus with other groups). **(B)** C57BL/6 mice (eight per group) were subcutaneously injected with TC-1 tumor cells (10^5^/mouse). Four days after tumor inoculation, mice were vaccinated with 2 μg of DNA vaccine three times at 5-day intervals. Therapeutic effects of the DNA vaccines were monitored from day 4 after inoculation. The line graph illustrates that the tumor volume in mice treated by BAFF-E7 DNA vaccine was significantly smaller than that of mice treated by the others (p<0.005, BAFF-E7 versus other groups). **(C)** Survival curve of the tumor-bearing mice treated by DNA vaccines. The results implied that BAFF-E7 DNA vaccine possesses preventive and therapeutic effects against TC-1 tumors and can sustain the survival of the treated mice longest. **(D)** C57BL/6 mice were injected with the same number of TC-1 cells and were vaccinated with 2 μg of DNA vaccine three times at 5-day intervals four days later after tumor inoculation. The 100μg of neutralizing antibody against CD8 T cells, CD4 T cells and NK cells were started to administer at the same day of first vaccination to the end of this assay with 2-day intervals. Mice without any treatment were set as control group. The results showed that administration of mouse CD8 neutralizing antibodies abrogated the anti-tumor effect, but not CD4 and NK neutralizing antibody (P<0.05 at day 13 and P<0.0001 at day 16). This implied CD8^+^ T cells contribute to the anti-tumor effect of BAFF-E7 DNA vaccine treatment. Error bar of each chart represents the standard error.*P<0.05, **P< 0.005, ***P<0.0001.

To determine the protective potential of chimeric BAFF–E7 DNA vaccine against tumors, we performed tumor protection assays in C57BL/6 mice inoculated with previously characterized E7-expressing TC-1 tumor model. C57BL/6 mice were intradermally vaccinated 3 times at 5-day intervals using a gene gun. Five days after the last vaccination, immunized mice were subcutaneously implanted with TC-1 cells. Mice were monitored for their tumor growth by measurement of tumor size. As shown in Figure 2A, mice vaccinated with chimeric BAFF–E7 remained tumor free after the TC-1 inoculation. By contrast, the other three groups of mice vaccinated with plasmids containing vehicle vector (pcDNA3.1), *BAFF* gene, or *E7* gene developed tumors. Chimeric BAFF–E7 DNA vaccine generated strongest TC-1 tumor rejection in mice.

To determine the therapeutic effect of chimeric BAFF–E7 DNA vaccine in treating TC-1 tumors, *in vivo* tumor treatment experiments were performed. C57BL/6 mice were first subcutaneously implanted with TC-1 cells. Four days after the tumor inoculation, mice were intradermally immunized (treated) by indicated vaccine three times at 5-day intervals through gene gun. As shown in Figure 2B, mice immunized with chimeric BAFF–E7 exhibited obvious inhibition of tumor growth on day 16 (P < 0.005, BAFF–E7 versus all other groups), and showed prolonged survival compared to those vaccinated with BAFF, E7, or pcDNA3.1 (Figure 2C; P < 0.005, BAFF–E7 versus all other groups).

In order to explore which effecter cells involved the antitumor effect of BAFF-E7 DNA vaccine, neutralizing antibodies target CD4, CD8, and NK 1.1 were administered to BAFF-E7 vaccinated and TC-1 tumor-bearing mice. As shown in Figure 2D, only anti-CD8 antibody abrogated the antitumor effect of BAFF-E7 vaccine (P=0.00405 at day 13 and P<0.0001 at day16, anti-CD8 antibody and control versus other groups). This result demonstrated that the antitumor effect of BAFF-E7 was through the effect of CD8^+^ T cells.

### Enhancement of E7-specific CD8^+^ T cell immunity induced by chimeric BAFF-E7 DNA vaccine is B-cell independent

Since BAFF is the factor for B cells activation and proliferation, it is reasonable to investigate whether the chimeric DNA vaccine can stimulated the production of anti-E7 antibody from vaccinated mice. The mice were immunized with indicated DNA vaccine three times at 5-day interval, and the serum were harvested one week after last vaccination. The existence of anti-E7 antibody in serum was detected by ELISA. The results demonstrated that all DNA vaccine cannot induce anti-E7 antibody production (Supplementary Figure 2). This implied anti-tumor effect of BAFF-E7 vaccine was not relative with anti-tumor antibody production.

**Figure 3:**
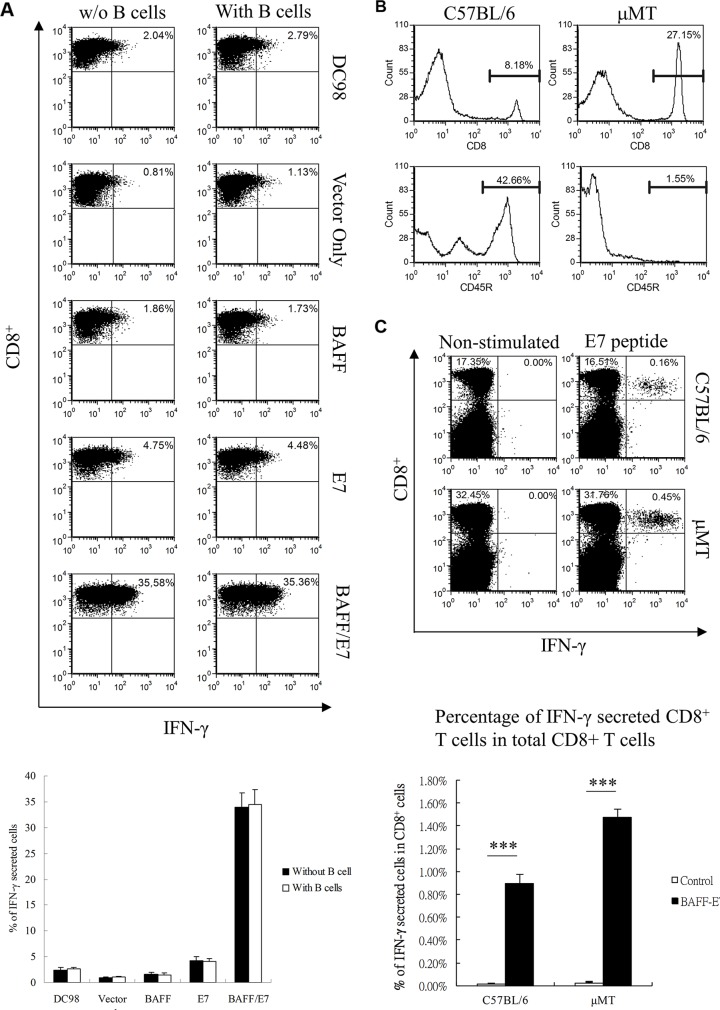
Role of B cells in the BAFF–E7 vaccine immune response **(A)** DC98 cells were transiently transfected with different DNA constructs using the Amaxa nucleofection system. One day after electroporation, 1 × 10^5^ transfected DC98 cells were co-cultured with or without 1 × 10^6^ C57BL/6 mouse B cells for another 24 h. At day 3, HPV-16 E7-specific T cells were added to the cell mixture with 1 μg/well of GolgiPlug. The proportion of IFN-γ-secreting cells was analyzed and depicted. Representative graph demonstrates that the presence of B cells did not significantly influence the activation of E7-specific CD8^+^ T cells. **(B)** Proportion of CD8^+^ and CD45R^+^ cells in the splenocytes from the C57BL/6 and μMT mice. only B cells, but not T cells, were lost in μMT mice. **(C)** C57BL/6 and μMT mice (five per group) were immunized with 2 μg of BAFF-E7 three times at 5-day intervals. Splenocytes from the mice were restimulated ex vivo with the E7 peptide and then characterized for E7-specific CD8+ T cells through analysis of intracellular IFN-γ staining cells. Splenocytes obtained from control and BAFF–E7 groups without peptide stimulation represented the background control. The percentage of E7-specific CD8^+^/IFN-γ^+^ double-positive T cells in all CD8^+^ T cells was shown as histogram. Representative graph shows that CTL-specific immunity can be generated by the chimeric BAFF-E7 DNA vaccine in both B-cell possessed or deficient mice (p<0.0001 BAFF-E7 versus control in wild type mice; p<0.0001, BAFF-E7 versus control in B cell deficient mice). Error bar in each chart represents the standard error. ***p < 0.0001.

We next tried to explore the potential mechanisms for the observed increase in E7-specific CD8^+^ T cells induced by chimeric BAFF–E7 DNA vaccinated mice. Since BAFF has been documented as a potent cytokine that stimulates B-cell maturation and survival, we hypothesized B cells might involve the direct augmentation of anti-tumor efficacy generated by BAFF-E7 DNA vaccine. Therefore, an *in vitro* cross-presentation assay was set up using DC98 cells transfected with BAFF-E7 DNA vaccines and cocultured with the E7-specific CD8^+^ T-cell line with B cells derived from C57BL/6 mice. As shown in Figure 3A, DC98 cells transfected with chimeric BAFF–E7 generated the highest number of CD8^+^/IFN-γ double-positive T cells. Nevertheless, the presence of B cells did not significantly influence the activation of E7-specific CD8^+^ T cells. To determine the role of B cells in antitumor immunity generated by the chimeric BAFF–E7 DNA vaccine *in vivo*, a tumor treatment experiment was performed in B-cell-deficient μMT mice. Figure 3B illustrates the proportion of B cells represented by the CD45R^+^ cells in the spleens of wild-type and μMT mice. B cell-deficient mice were first subcutaneously implanted with TC-1 cells. Four days after the tumor inoculation, these mice were intradermally treated with indicated vaccines three times at 5-day intervals using a gene gun. We analyzed the CD8^+^-restricted immune response through intracellular cytokine staining for assessing IFN-γ secreted by freshly isolated splenocytes (restimulated with the E7 peptide) 3 days after the final vaccination. Figure 3C shows representative flow data depicting both wild-type and B cell deficient mice vaccinated by the chimeric BAFF-E7 DNA vaccine showing significantly increased frequencies of E7-specific CD8^+^/IFN-γ^+^ double-positive T cells. Collectively, these data suggest that the presence or absence of B cell did not affect the cytotoxic T-lymphocyte (CTL)-specific immunity generated by the chimeric BAFF–E7 DNA vaccine.

### BAFF-E7 DNA vaccine does not alter the maturation pattern of dendritic cells

The basic mechanism of DNA vaccine was in enhancing the introduction of antigens to dendritic cells and then presenting to effectors cells. Because the maturation of DC is associated with the efficiency of antigen presentation, the effect of BAFF-E7 DNA vaccine on the maturation of DC cells is worthy of being explored. DC98 cells were transfected with different recombinant constructs representing respective DNA vaccine through electro-transfection. The maturation of DC98 cells was further determined by the expression of CD40, CD80, and CD86 using flow cytometry. The results demonstrated that the surface expression level of CD40, CD80 and CD86 of all groups were not changed (Supplementary Figure 3). This implied that the effect of BAFF-E7 DNA vaccine was not through alteration of DC maturation.

### Fusion of BAFF to HPV-16 E7 protein targets E7 into the ER

**Figure 4:**
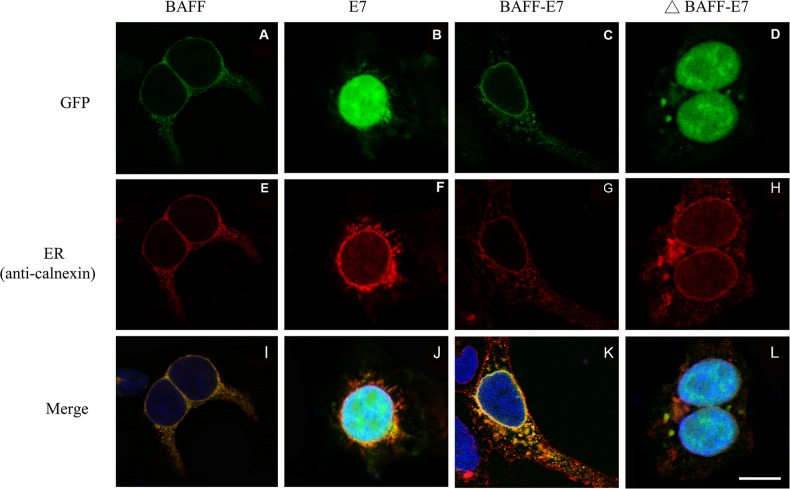
Distribution of BAFF, E7, BAFF-E7, and ΔBAFF-E7 proteins within cells The 293T cells were transfected with expression clones of BAFF **(A, E**, and **I)**, E7 **(B, F**, and **J)**, BAFF-E7 **(C, G**, and **K)**, and ΔBAFF-E7 **(D, H**, and **L)** fused with GFP. Localization of the ER and nucleus was determined by staining the cells with PE-labeled anti-calnexin antibody and Hoechst 33342. Fluorescent images were acquired using a confocal microscope. DNA constructs are shown at the top of the figures. (A–D) Distribution of different constructs of GFP fusion proteins; (E–H) localization of the ER; (I–L) merged images. Representative figure demonstrated that fusing the full-length BAFF to E7 targets the chimeric protein into ER, whereas transmembrane-domain truncated BAFF abrogates this phenomenon. The size of the scale bar was 10 μm.

BAFF is a type II membrane protein, which is anchored with a transmembrane domain to the lipid membrane and has its C-terminal domain targeted to the ER lumen during synthesis. Therefore, we anticipated that fusing E7 to the C-terminal of BAFF might facilitate antigen presentation by delivering peptides into the ER and directly enhance the MHC class I presentation of E7 peptides in BAFF–E7-expressing cells, while the trunked BAFF (ΔBAFF) lacking 128 N-terminal residuals, including transmembrane domain, may lost this function. To determine whether the fusion to BAFF influences the subcellular localization of E7, we generated several GFP tagged DNA constructs (BAFF–GFP, E7–GFP, BAFF–E7–GFP, and ΔBAFF–E7–GFP) by employing the pEGFP-N1 vector. Confocal fluorescent microscope revealed a nuclear distribution of the cells transfected with E7–GFP (Figure 4B, 4F, and 4J) or ΔBAFF–E7–GFP (Figures 4D, 4H, and 4L). In contrast, cells transfected with BAFF–GFP (Figures 4A, 4E, and 4I) or BAFF–E7–GFP (Figures 4C, 4G, and 4K) presented a network pattern consistent with ER localization. To further confirm whether BAFF–GFP and BAFF–E7–GFP were distributed to the ER, an antibody against calnexin, a well-characterized marker for the ER was also used. As shown in Figure 4I and 4K, colocalization with calnexin was observed in the cells transfected with the BAFF–GFP or BAFF–E7–GFP construct but not in those transfected with E7–GFP or ΔBAFF–E7–GFP. These data indicate that fusing the full-length BAFF to E7 facilitates the targeting of the chimeric protein into the ER, and truncating the BAFF transmembrane domain abrogates this phenomenon.

### Fusion of BAFF lacking 128 N-terminal residues to HPV-16 E7 protein failed to generate E7-specific CD8^+^T-cell immune response in vaccinated mice

**Figure 5:**
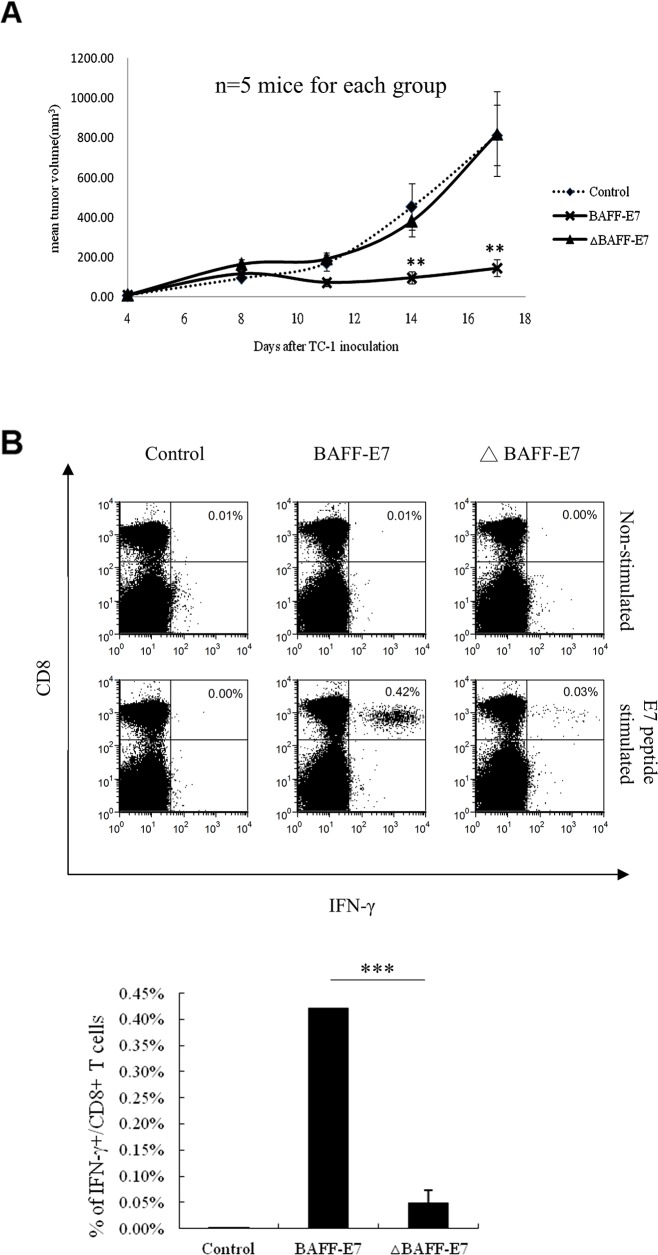
Antitumor effect of the BAFF-E7 and ΔBAFF-E7 DNA vaccines *in vivo* **(A)** C57BL/6 mice (five per group) were first subcutaneously injected with TC-1 tumor cells (10^5^/mouse). Four days after tumor inoculation, mice were vaccinated with 2 μg of DNA vaccine per mouse, three times at 5-day intervals. Tumor volume was measured starting from day 4 after tumor inoculation. The line graph illustrates that the ΔBAFF-E7 DNA vaccine cannot significantly inhibits the growth of TC-1 cells (Day 14, p<0.005, BAFF-E7 versus ΔBAFF-E7; day 17, p<0.005, BAFF-E7 versus ΔBAFF-E7). **(B)** Splenocytes from five mice in each group were pooled and restimulated ex vivo with the E7 peptide and then characterized for E7-specific CD8^+^ T cells through analysis of intracellular IFN-γ staining cells. Splenocytes from the control, BAFF, E7, and BAFF–E7 groups without peptide stimulation served as the background control. Representative graph shows that ΔBAFF-E7 DNA vaccine did not induce high percentage of E7-specific CD8^+^ T cells as BAFF-E7 did. (p=0.0004, BAFF-E7 versus ΔBAFF-E7). The result indicates that the property of ER-accumulation by fusing E7 to the full length of BAFF protein is critical for E7-specific CD8^+^ CTL activation and antitumor effects. Error bar in each chart represent standard error. **p < 0.005; ***p< 0.0005.

To investigate whether passing through the ER–Golgi pathway is crucial for the significant E7-specific DNA vaccine, we compared the antitumor effect and E7-specific immune response between wild type BAFF-E7 and ΔBAFF–E7 construct. *In vivo* tumor treatment experiment was performed as previously described. Figure 5A shows that mice vaccinated with the ΔBAFF–E7 DNA vaccine did not demonstrate inhibition in the growth of TC-1 tumors. Furthermore, intracellular cytokine staining of the splenocytes from ΔBAFF–E7-vaccinated mice did not show enhancement of E7-specific CD8^+^ T cells (Figure 5B). These results suggested that the property of ER accumulation by fusing E7 to the BAFF protein was critical for E7-specific CD8^+^ CTL activation and antitumor effects.

## DISCUSSION

DNA vaccines can be easily manipulated and are cost effective considering their manufacturing and storage; therefore, their use has become a major trend in clinical applications for cancer treatment. Compared with toxic attenuated pathogens, recombinant proteins, or whole tumor cells, DNA vaccines are relatively easy to be qualified. Moreover, DNA vaccines have been shown to not induce anti-DNA antibodies, even when multiple immunizations are administered. However, the low immunogenicity induced by DNA vaccines hampers their clinical application in large animals and humans. Implementing various DNA vaccine designs to enhance the immune response has attracted much attention in this field. In our study, a BAFF–E7 fused DNA vaccine has been proved to be effective in E7-expressed cancer prevention and therapy. By forcing the E7 antigen locating in the endoplasmic reticulum (ER) lumen, BAFF enhanced MHC class I presentation and activated more E7-specific CD8^+^ T cells, resulting in a superior antitumor effect.

Cancer cells, in contrast to most infectious diseases, are killed by cellular immune responses. Tumor antigens are often weakly immunogenic and tolerated in the patients [28]. In order to induce potent cellular immune responses against cancer growth and even eradicate tumor cells, an effectively designed DNA vaccine is required. BAFF has been documented to enhance immune responses by stimulating B-cell proliferation and maturation [21]. However, coadministering a HIV-1 DNA vaccine with a surfactant protein D-fused trimeric BAFF DNA vaccine considerably induced Gag-specific IL-2 secretion in memory T cells but not anti-Gag antibody responses. In this study, BAFF–E7 fusion DNA vaccine strongly stimulated E7-specific CD8^+^ T cells, and the presence of B cells did not influence the enhancement. Overall, these results reveal that B cells did not play a major role in the immune response generated by BAFF–E7 fusion DNA vaccines and indicate that mechanisms other than B-cell activation may be responsible for the specific cytotoxic T-cell immune response of BAFF–antigen fusion DNA vaccines.

Alter the pattern of dendritic cells maturation is a considered strategy for design of cancer vaccine. There are a variety of molecules that can be delivered as DNA vaccine to improved antigen presenting cell activity, like chemokine receptors (e.g. CCR1, CCR5, and CCR6), Toll-like receptors (e.g. TLR2, TLR3, and TLR6), and TNF-family receptors (e.g. Fas, CD40, and OX40L) [6]. Fusion of HIV Gag protein with CD40L was demonstrated to enhance DC maturation in mouse model and induced CD8^+^ T cell response [29]. In this study, however, BAFF-E7 chimerical DNA vaccine did not alter the maturation pattern of DC. The results demonstrated that the effect of CTL activation of BAFF-E7 DNA vaccine may through other mechanisms.

Forcing target epitopes into the ER have been reported in enhancing immunogenicity through acting like a genetic adjuvant [30, 31]. A study showed that fusing an adenovirus E3 leader sequence with a mutated p53 sequence increased the CTL activity and protected from a mutated p53-expressed tumor inoculation [32]. In addition, DNA vaccine-encoded secreted or membrane-bound ovalbumin generated stronger CTL responses than cytoplasmic ovalbumin did [33]. In this study, truncated BAFF lost its ER transportation ability and thus the function of the immune intensification by the antigen-fused DNA vaccine and the antitumor effect was abrogated. All these results are compatible with the theory that ER entry enhances MHC class I-specific immune responses. However, a previous study reported that DNA vaccine with deleted signal peptide of human carcinoembryonic antigen (CEA) can increase the cellular retention time and strongly suppressed tumor growth in a human CEA-expressing colon cancer cell model [34]. This conflict indicates that the major effect of these vaccines originates from the enhanced antigen processing and MHC class I presentation through different mechanisms. We speculate that different antigen types might require different strategies of antigen manipulation. For cellular proteins, ER entry may increase the frequency of epitope-MHC class I molecule contact and enforce antigen presentation. For secretary or membrane-bound proteins, prolonging the intracellular retention of such antigens by deleting the leading sequence increases the probability of antigen processing through lysosomes and strengthens MHC class I presentation. Hence, DNA vaccines for various antigens must be designed using different approaches according to the nature of the targets.

MHC class I presentation depends on antigen processing in proteasomes and antigen translocation into the ER through the Tap protein to load onto MHC class I molecules. Although BAFF can facilitate the ER entry of fused antigen, it does not respond to antigen processing. A previous study revealed that antigenic peptides were processed through a retrograde transfer from the ER to the cytosol [35]. Another study on the ER-resident protein JAW1 showed that JAW1 can facilitate the MHC class I-mediated presentation of antigens fused at their C termini in Tap-deficient cells. Furthermore, the inhibition of proteasomes did not affect the release of a JAW1-fused peptide [36]. It indicates the existence of an unusual mechanism underlying peptide entry into the class I presentation pathway. In addition, the trans-Golgi network protease furin was reported to facilitate endogenous viral peptide presentation by the MHC class I system in a TAP-independent pathway [37]. Our data reveal that the truncated BAFF–E7 vaccine with defect of the furin cleavage site located between the transmembrane domain and TNF homology domain lost the ability of CTL activation and tumor growth inhibition. It is possible that furin protease process is the other mechanism accounting for the immune enhancement by BAFF-E7 fusion DNA vaccine.

The primary step in CTL activation does not require the participation of B cells in wild-type and B-cell-deficient mice infected with lymphocytic choriomeningitis virus [38]. However, the T-cell death phase was more pronounced in B-cell-deficient mice, which resulted in lower absolute numbers of virus-specific CTL. This observation may explain the result that although the BAFF–E7 DNA vaccine stimulates a comparable proportion of E7-specific CTL in the splenocytes of B-cell-deficient mice, the therapeutic effect was not as good as performing in wild type mice. (Supplementary Figure 4).

In conclusion, the BAFF–E7 fused DNA vaccine effectively prevented and inhibited the growth of HPV E7-expressing tumors. B cells were not engage in the process of antigen presentation and CTL activation, while the transmembranous domain and furin cleavage site of BAFF might involve the antigen processing and enhance BAFF-fused antigen presentation through the MHC class I pathway. The results demonstrate the preventive and therapeutic effectiveness of the DNA vaccine in cancer treatment. The presented vaccine characteristics of ease of large-scale preparation, stability for storage and distribution, fewer side effects imply the value of such a vaccine construction.

## MATERIALS AND METHODS

### Mice and murine tumor cell lines

C57BL/6 and μMT mice (strain: B6._129_S2–Ighm^tm1Cgn^/J) were purchased from BioLASCO, Taiwan. The animals were housed under specific pathogen-free conditions. The mouse non-small cell lung cancer cell line TC-1 is C57BL/6 origin and an engineered expression of HPV-16 E7. E7-specific CD8^+^ T cell line, and DC98 mouse immortalized dendritic cell line used in this study have been kindly provided by Dr. T-C Wu [39]. All cells were all cultured in a RPMI 1640 medium (Gibco, Grand Island, NY) supplemented with 10% fetal bovine serum (FBS; Biological Industrial, Israel), 1 mM of 2-mercaptoethanol(Gibco, Grand Island, NY), 100 U/mL of penicillin (Gibco, Grand Island, NY), and 100 pg/mL of streptomycin (Gibco, Grand Island, NY) in a humidified atmosphere of 5% CO_2_/95% air at 37°C.

### Plasmid DNA construction

**Figure 6:**
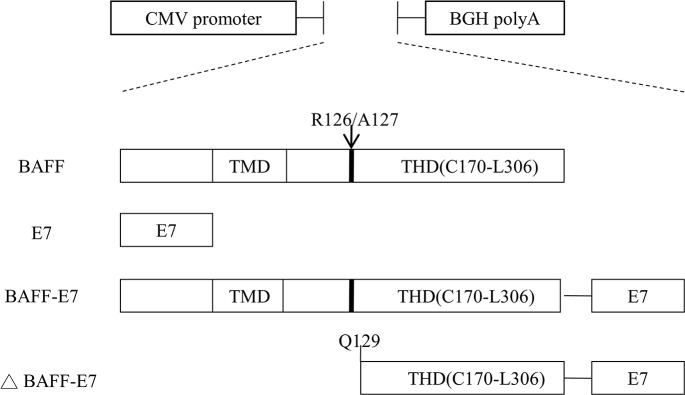
Schemes of the DNA vaccine constructs HPV-16 E7 gene and full length of the *BAFF*, *BAFF-E7*, and Δ*BAFF–E7* genes were inserted under the control of the CMV promoter in the pcDNA3.1 vector. TMD: transmembranous domain. THD: TNF homology domain. R126/A127: furin cleavage site.

The mouse pcDNA3–BAFF, pcDNA3–E7, and pcDNA3–BAFF–E7 plasmids were cloned by us in the lab. The ΔBAFF–E7 DNA fragment was constructed using a pcDNA3–BAFF–E7 plasmid as the template and polymerase chain reaction (PCR) amplification with a forward primer (5′-AAATTTCTCGAGGCCACCATGCAGGGACCAGAGGAAAC-3′) and reverse primer (5′-AAATTTGGATCCGCCTGAGAACAGATGGGGCA-3′). The PCR product was double digested with XhoI/BamHI and subcloned back to the pcDNA3 vector. The sBAFF DNA fragment was constructed using the same template with a forward primer and reverse primer. The PCR product was double digested with EcoRI/BglII and subcloned into pFUSE-mIgG2A-Fc2 vector (Invivogen, San Diego, CA). Figure 6 shows the scheme of different DNA vaccine constructs. Green fluorescent protein (GFP) fusions were generated by subcloning all DNA fragments into pEGFP–N1 (Takara Bio USA, Inc., Mountain View, CA).

### DNA vaccination

All the prepared vaccine plasmids were endotoxin free (Qiagen, Hilden, Germany). DNA-coated gold particles were administered using a helium-driven gene gun (BioRad Laboratories Inc., Hercules, California, USA) according to the manufacturer's instructions. The control plasmid, E7, BAFF, BAFF–E7, or ΔBAFF–E7 DNA-coated gold particles were delivered to the shaved abdominal region of mice with a discharge pressure of 400 psi of gene gun.

### Isolation of tumor-infiltrating lymphocytes

Tumors from the DNA vaccine-treated mice were dissected and chopped into 2–3-mm pieces using a razor blade. One gram of tumor pieces was then incubated with 10 mL of an enzyme mixture dissolved in RPMI 1640 with 2% FBS [collagenase type I (0.05 mg/mL), collagenase type IV (0.05 mg/mL), hyaluronidase (0.025 mg/mL), and DNase I (0.01 mg/mL), all from Sigma, St. Louis, MO] for 15 min at 37°C. After a brief centrifugation process, the cells were resuspended in a fresh enzyme mixture and incubated for another 15 min at 37°C with gentle shaking. The undigested materials were filtered using 100-μm cell strainers, and those non-adherent cells were recovered and washed through centrifugation in a complete medium.

### Intracellular cytokine staining and flow cytometry

The mice were immunized with 2 μg of various DNA vaccine constructs and received a boost dose at 1-week interval for total 3 doses. Splenocytes were harvested 1 week after the final vaccination. Before the execution of intracellular cytokine staining, 1 × 10^7^ of pooled splenocytes or 2 × 10^6^ of tumor-infiltrating lymphocytes from each vaccinated group were incubated for 16 h with 1 μg/mL of the HPV16-E7 peptide (a.a. 49–57) containing an MHC class I epitope and 1 μL/mL of GolgiPlug (BD bioscience, San Diego, CA) to detect E7-specific CD8+ T cells. Those cells were first stained with APC-conjugated anti-mouse CD8a antibody (eBioscience, San Diego, CA). After fixation and permeabilization with perm/wash buffer (BD bioscience, San Diego, CA), cells were further stained intracellularly with fluorescein-isothiocyanate-conjugated anti-interferon-γ (INF-γ) antibody (eBioscience, San Diego, CA). The proportion of CD8+/IFN-γ+ lymphocytes were analyzed by flow cytometry (Calibur, BD Bioscience, San Diego, CA).

### *In vivo* tumor protection

Twenty female C57BL/6 mice were divided into four groups. Two micrograms of pcDNA3–BAFF, pcDNA3–E7, or pcDNA3–BAFF–E7 DNA vaccine constructs were administered to each group of mice using a gene gun three times at 5-day intervals. Untreated mice were set as the control. Five days after the final vaccination, 1 × 10^5^ TC-1 cells were subcutaneously injected into each mouse. Tumor growth and sizes were measured twice a week as the longest length × width × height.

### *In vivo* tumor treatment

In the tumor treatment setting, 1 × 10^5^ TC-1 cells were subcutaneously inoculated into 32 C57BL/6 mice. Four days later, mice were divided into four groups and subjected to 2 μg of DNA vaccination through gene gun. Tumor size was measured as described previously. A mouse was considered dead when the diameter of bearing tumor is up to 20 mm.

### *In vitro* assay of cross presentation

A mouse dendritic cell line, DC98, was electrotransfected with pcDNA3, pcDNA3–BAFF, pcDNA3–E7, and pcDNA3–BAFF/E7 through nucleofection (Lonza Cologne AG, Cologne, Germany) and cultured for further 48 hours. Subsequently, 1 × 10^5^ transfected DC98 cells were cocultured with 5 × 10^5^ E7-specific T cells (derived from C57BL/6) with or without B cells derived from C57BL/6 mouse in a complete medium with 1 uL/mL of GolgiPlug (BD Bioscience, San Diego, CA). Intracellular trapped INF-γ was stained with a fluorescent antibody and assayed through flow cytometry, as described previously.

### Immunofluorescence cytostaining

Briefly, 1 × 10^5^ 293T cells were suspended in 200 μL of complete medium and seeded onto a cover slip within a 6-well plate. Six hours later, 2 mL of the complete medium was added to each cell-containing well and cells were left cultured overnight at 37°C. On the next day, 3 μg of pEGFP–BAFF, pEGFP–E7, pEGFP–BAFF–E7, and pEGFP–ΔBAFF–E7 were transfected into each well with a jetPEI reagent (Polyplus-transfection, Illkrich, France) and kept cultured for 2 days. Each set of cells were fixed with 4% paraformaldehyde and stained by a fluorescent anticalnexin antibody (eBioscience, San Diego, CA). After counterstaining with Hoechst 33342, the cell-attached cover slips were mounted with a fluorescent mounting medium (Dako, Glostrup, Denmark) and observed through a confocal microscope.

### Statistics

All results are presented as mean ± standard error (SE) from at least two independent experiments. Comparisons between different data points were performed using the Student t test and one way ANOVA test.

## SUPPLEMENTARY MATERIALS FIGURES AND TABLES


