# Supplementary material for: Enhanced anti-tumor therapeutic efficacy of DNA vaccine by fusing the E7 gene to BAFF in treating human papillomavirus-associated cancer
Source: Oncotarget. 2017 Mar 9;8(20):33024–36. doi: 10.18632/oncotarget.16032 (PMC5464847; doi:10.18632/oncotarget.16032)
Supplement: Supplementary file 1 [file oncotarget-08-33024-s001.pdf]

Enhanced anti-tumor therapeutic efficacy of DNA vaccine by fusing the E7 gene to BAFF in treating human papillomavirus-associated cancer

SUPPLEMENTARY FIGURES

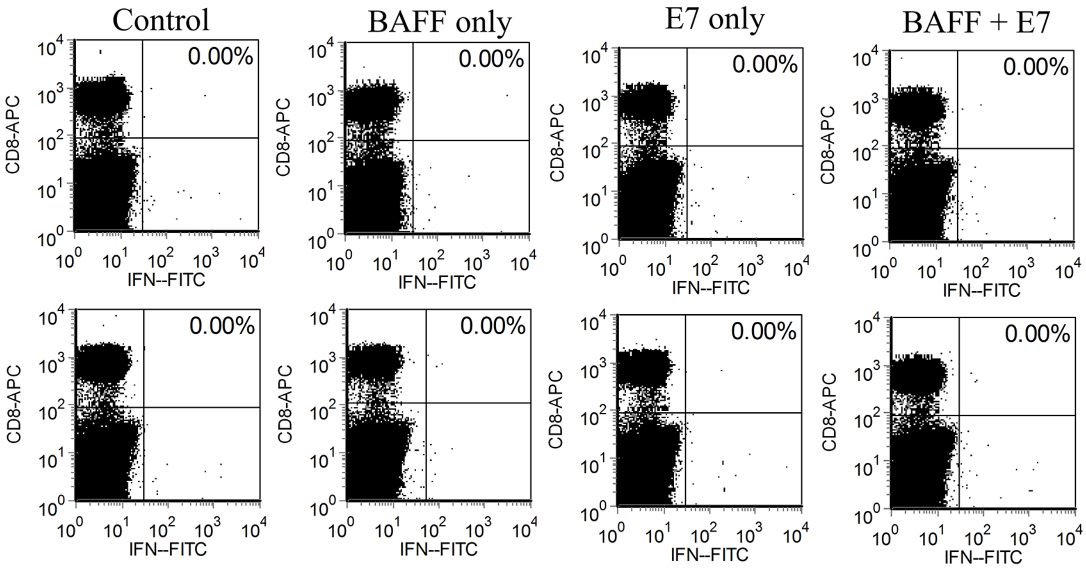

Supplementary Figure 1: Characterized CD8+ T cell-immune response of the BAFF, E7, or BAFF/E7-mixed DNA vaccine. C57BL/6 mice were immunized with 2 μg of BAFF, E7, or BAFF/E7-mixed DNA vaccine using a gene gun for total three times at 5-day intervals. Untreated mice served as the control. One week after last immunization, splenocytes harvested from different groups of mice were restimulated ex vivo with HPV16 E7 peptide and characterized for E7-specific $CD8^+$ T cells through flow analysis of intracellular IFN-γ staining cells. Splenocytes without peptide stimulation represented the background control. The results demonstrated that all DNA vaccine cannot stimulate E7-specific $CD8^+$-T cells immune response.

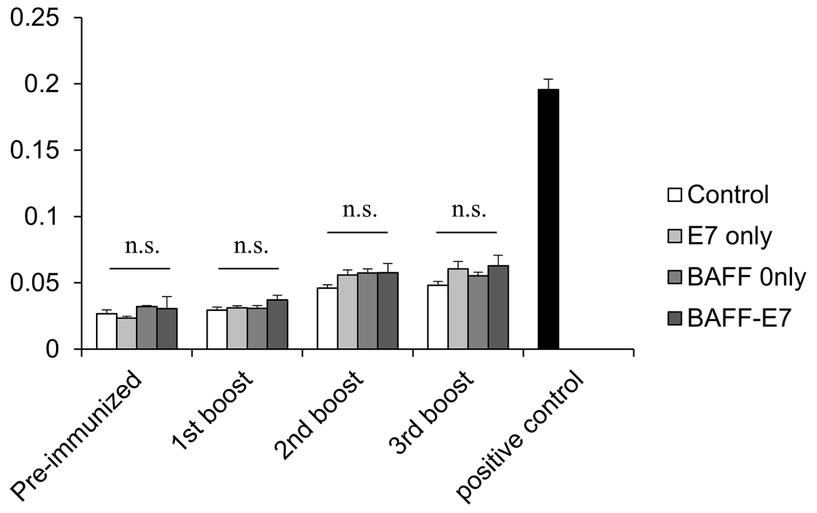

Supplementary Figure 2: Effect of BAFF-E7 DNA vaccine on anti-E7 antibody production. C57BL/6 mice were immunized with 2 μg of BAFF, E7, or BAFF-E7 DNA vaccine using a gene gun for total three times at 5-day intervals. Untreated mice served as the control. Serum was harvest from each mice before immunization (Pre-immunized) and four days after each time of immunization. The titers of anti-E7 antibody in mouse serum from each mouse were detected by ELISA assay. Compared with non-immunized mice, there was no significant difference in titers of anti-E7 antibody in all groups, which implies that BAFF-E7 vaccine is not effective in antibody induction.

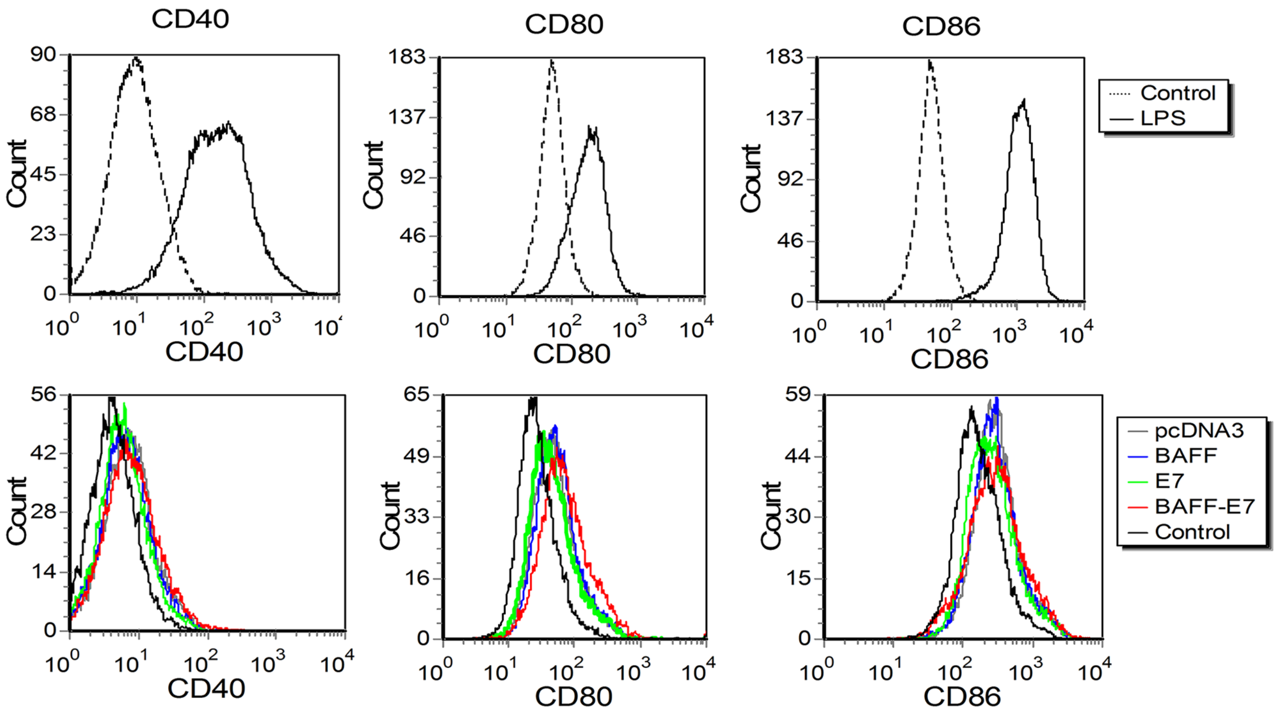

Supplementary Figure 3: Effect of BAFF-E7 DNA vaccine on dendritic cell maturation. Two micrograms of DNA vaccine were separately electro-transfected into 2.5×10^5 of DC 98 cells. Three days later, cells were stained with fluorescence-conjugated anti-CD40, anti-CD80, or anti-CD86 antibodies. Maturation of DC 98 cells was determined by flow analysis. Cells treated with 1μg/mL of LPS served as the positive control. The results demonstrated that BAFF, E7 and BAFF-E7 DNA vaccine did not alter the pattern of surface expression of CD40, CD80, and CD86 molecules. It implies that vaccine treatment might not involve the maturation of dendritic cells.

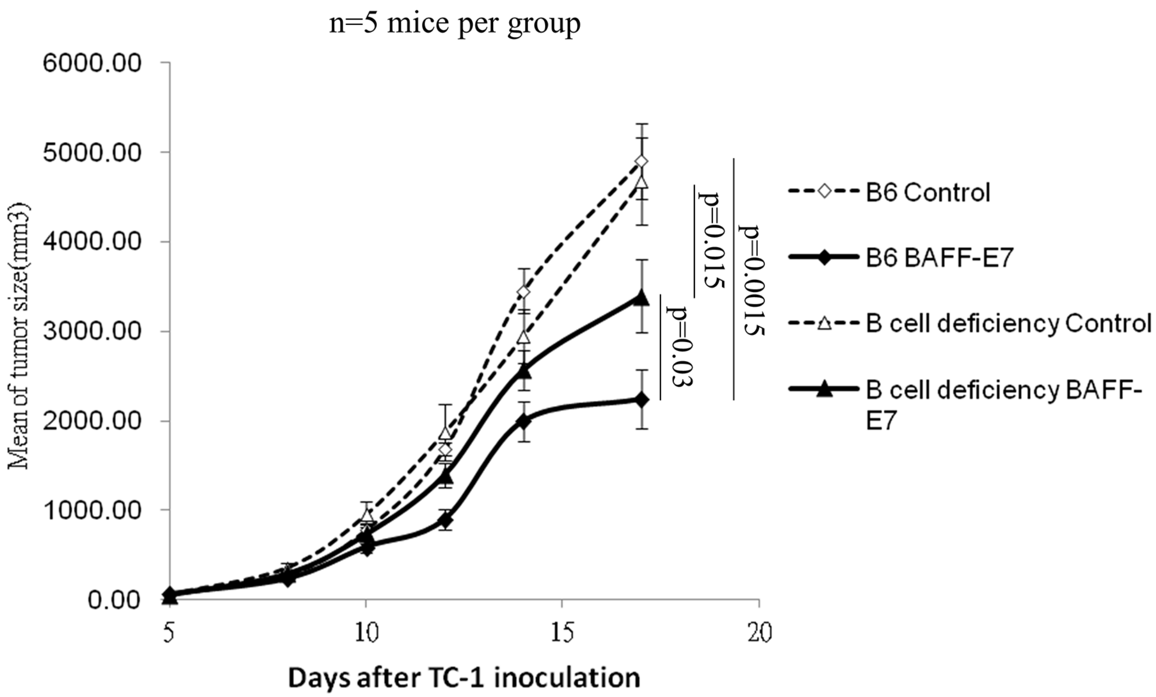

Supplementary Figure 4: Therapeutic effect of the BAFF-E7 DNA vaccine in B-cell-deficient mice. C57BL/6 mice and μMT mice were subcutaneously injected with TC-1 tumor cells (10^5/mouse). Four days after tumor inoculation, mice were vaccinated with 2 μg of all DNA vaccines three times at 5-day intervals. Therapeutic effects of the DNA vaccines were monitored by measuring the tumor growth starting from day 4 after tumor inoculation. BAFF-E7 vaccine in both wild type and B-cell deficient mice could effectively inhibit the tumor growth (p= 0.0015 and 0.015, control versus BAFF-E7). However, the anti-tumor effect in B-cell deficient mice is not as good as that in wild-type mice (p=0.03, B6 BAFF-E7 versus B cell deficiency BAFF-E7).
